# Adaptive Dual-Beam Tracking for IRS-Assisted High-Speed Multi-UAV Communication Networks

**DOI:** 10.3390/s25216757

**Published:** 2025-11-05

**Authors:** Zhongquan Peng, Guanglong Huang, Qian Deng, Xiaopeng Liang

**Affiliations:** 1 School of Intelligent Manufacturing, Jiangxi Institute of Applied Science and Technology, Nanchang 330100, China; zhongquanpeng@163.com; 2 College of Electronics and Information, Hangzhou Dianzi University, Hangzhou 310018, China; 3 Haikou Bureau of Science, Technology, Industry and Informatization, Haikou 570100, China; 4 School of Information and Communication Engineering, Hainan University, Haikou 570228, China; dqian108@163.com (Q.D.); liangxiaopeng@hainan.edu.cn (X.L.)

**Keywords:** UAVs communication network, IRS, max-min optimization problem, beam tracking

## Abstract

This study investigates the communication network (MUAVN) of intelligent reflecting surface (IRS)-assisted high-speed multiple unmanned aerial vehicles, considering that highly dynamic UAVs may incur poor performance due to severe channel fading and rapid channel changes. Our objective is to design an adaptive dual-beam tracking scheme that mitigates beam misalignment, enhances the performance of the worst-case UAV, and sustains reliable communication links in the high-speed MUAVNs (HSMUAVNs). We first exploit an attention-based double-layer long short-term memory network to predict the spatial angle information of each UAV, which yields optimal beam coverage that matches to the UAV’s actual flight trajectory. Then, a worst-case UAV’s received beam components signal-to-interference plus noise ratio (SINR) maximization problem is formulated by jointly optimizing ground base station’s beam components and IRS’s phase shift matrix. To address this challenging problem, we decouple the optimization problem into two subproblems, which are then solved by leveraging semi-definite relaxation, the bisection method, and eigenvalue decomposition techniques. Finally, the adaptive dual beams are generated by linearly weighting the obtained beam components, each of which is well-matched to the corresponding moving UAV. Numerical results reveal that the proposed beam tracking scheme not only enhances the worst-case UAV’s performance but also guarantees a sufficient SINR demanded across the entire HSMUAVN.

## 1. Introduction

Unmanned aerial vehicles (UAVs) play a paramount role in wireless communication networks due to their low cost, high maneuverability, and on-demand deployment capabilities [1,2,3,4]. However, providing stable and reliable communications for multiple high-speed UAVs remains a significant challenge. Especially for edge UAVs, the instability of beam gain is more pronounced due to severe channel fading and rapid changes [5]. Existing beam tracking schemes are fragile in highly dynamic environments, and the acquired channel state information (CSI) becomes obsolete almost instantaneously. Therefore, it is imperative to investigate the beam tracking schemes that dynamically adapt to high mobile UAVs to promptly establish stable links in high-speed multi-UAV networks (HSMUAVNs).

### 1.1. Related Works

Recently, UAVs have been seamlessly integrated into a broad spectrum of industries, spanning agriculture, survey mapping, vehicular communication, disaster response, and so on. Considering that task-driven UAVs operate at high velocities, maintaining accurate beam tracking presents substantial challenges in practical scenarios [6,7,8,9]. Firstly, the direct tracking of UAVs’ direction-of-arrival (DoA) and direction-of-departure (DoD) incurs inherent latency due to UAV mobility and dynamic perturbations, resulting in beam misalignment and degraded link performance; secondly, employing beam training for CSI estimation leads to extensive feedback overhead, inherent estimation errors, and increased resource consumption; thirdly, existing narrow beam tracking methods face challenges in maintaining stable and robust beam gain. Intelligent reflecting surfaces (IRSs) possess the ability to dynamically reconfigure the wireless environment [10,11,12]. Therefore, integrating IRS into high-speed multi-UAV networks (HSMUAVNs) brings several notable advantages which include (1) compensating the high path loss and building blockages; (2) canceling interferences among UAVs; (3) steering the signal toward areas that are challenging to cover and improving worst-case UAV performance. However, scant attention has been paid to IRS-assisted beam tracking in high dynamic scenarios which generates high angular speeds. In [13], the roadside IRS and vehicle-side IRS beam tracking strategies were proposed in high-mobility vehicle communications, and a performance comparison between these two practice IRS deployment schemes was provided. However, these strategies were tailored to vehicular networks, whose channel variations are significantly slower than those induced by UAVs. Hence, they may prove vulnerable in HSMUAVNs, as the acquired CSI rapidly becomes obsolete. In [8], an integration of sensing and communications (ISAC)-assisted beam tracking scheme in multipath channels is proposed, but it came at the cost of beam training time. In [14], the geometry dynamic channel model and a deep learning-based channel narrow beam tracking method with small pilot overheads in IRS-assisted single UAV communication were proposed; however, this work overlooks beam misalignment inherent in highly dynamic UAV scenarios. As an alternative to the existing narrow beam tracking, angle-aware beam tracking schemes have been considered to reach beam alignment for UAV-GBS links. Ref. [15] pointed out that beam coverage is a key factor in beam reconstruction for high-speed UAV networks and proposed an efficient beam alignment framework integrating channel tracking and beamforming design, but it did not involve IRS and did not fully consider the severe channel fading and rapid channel changes for edge users in HSMUAVNs.

### 1.2. Motivations and Contributions

Unlike the existing works, we highlight how designing an adaptive dual-beam pattern based on the predicted spatial angle information (SAI) reduces the beam misalignment in HSMUAVN scenario. Moreover, the proposed adaptive dual-beam tracking simultaneously boosts the worst-case UAV’s performance and maintains a reliable communication link across the entire HSMUAVN. The main contributions of this paper include the following:

(1) The attention-based double-layer long short-term memory (DL-LSTM) neural network is exploited to effectively predict the SAI of each high-speed UAV for the next frame. Therefore, the knowledge of each UAV’s trajectory or angular transition model is not required. Then, the optimal beam coverage (BC) and the number of beam components for each UAV can be efficiently estimated based on the predicted SAI, which fully considered the prediction errors, the angular process noise, and the high-speed UAV motion, and always supports the coverage of high-speed UAVs within a frame.
(2) To enhance the edge UAV’s performance and maintain a reliable communication link in HSMUAVNs, a worst-case UAV’s received beam components signal-to-interference plus noise ratio (SINR) maximization problem is formulated by jointly optimizing GBS’s beam components and IRS’s phase shift matrix (PSM). Furthermore, the formulated non-convex problem is effectively tackled by an iterative algorithm based on semi-definite relaxation, the bisection method, and eigenvalue decomposition techniques.
(3) Based on the acquired optimal BC and beam components, we design an adaptive dual-beam tracking scheme which is generated by linearly weighting these beam components. Simulation results validate that the proposed adaptive dual-beam tracking scheme improves both the achievable rate and robustness of the worst-case UAV in IRS-assisted HSMUAVNs.

### 1.3. Organization and Notations

In Section 2, we propose the IRS-assisted HSMUAVN system model. In Section 3, we design an adaptive dual-beam tracking algorithm and formulate the worst-case UAV’s received beam components SINR maximization problem. Furthermore, we provide the complexity analysis. The simulation results and the conclusion are presented in Section 4 and Section 5, respectively.

*Notations:* Lower-case, bold-face lower-case and bold-face capital letters represent scalars, vectors, and matrices, respectively. ∥·∥ signifies the Euclidean norm, while |·| represents the absolute value. Operation symbol ⊗ represents two vectors’ element-wise multiplication. Finally, $\mathrm{CN}\left(\mu ,\sigma^{2}\right)$ represents a random variable that follows the circularly symmetric complex Gaussian distribution with μ mean and $\sigma^{2}$ variance.

## 2. System Model

**Figure 1:**
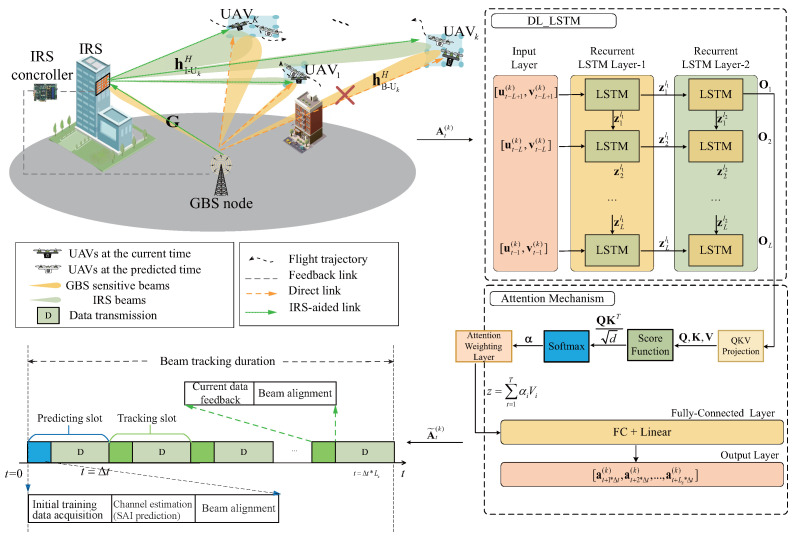
Adaptive dual-beam tracking scheme for IRS-assisted HSMUAVNs.

As shown in Figure 1, the considered IRS-assisted HSMUAVNs consist of *K* single-antenna moving UAVs, a building-mounted IRS equipped with $N_{r}=N_{rx}\times N_{rz}$ passive reflective elements, a GBS with $N=N_{x}\times N_{z}$ antennas, and $M=M_{x}\times M_{z}$ radio frequency chains. Moreover, a uniform planar array (UPA) is adopted at both the BS and the IRS [16,17]. Specifically, the GBS communicates with *K* high-speed UAVs with the aid of IRS. In the considered HSMUAVNs in Figure 1, the duration of the adaptive dual-beam tracking scheme includes a predicting slot (PS) and $L_{s}$ tracking slots (TS). The PS mainly consists of an initial training data acquisition block, a channel estimation block, a beam alignment block, and a data transmission block, while the TS involves the current data feedback block, a beam alignment block, and a data transmission block. The initial training data acquisition block serves as a preparatory step prior to establishing reliable GBS-to-UAV links. Next, the channel estimation block adopts a deep learning-based method to accurately estimate the SAIs of each UAV node without invoking any beam sweeping procedure. Here, a time slot is denoted by ∆t and the length of a beam tracking duration is denoted by $∆t*L_{s}$. As a matter of fact, because IRSs and UAVs are usually located at high altitudes, there exists of line-of-sight (LoS) paths between IRS-related and UAV-related channels. Thus, the GBS-IRS, GBS-UAVs, and IRS-UAVs links can be modeled as reasonable Rician fading channels [18]. Thus, the channel gain of the GBS-IRS link is given by

(1) $$\begin{array}{cc} \; & G=\sqrt{\rho_{B-R}d_{B-R}^{-\Gamma_{B-R}}\frac{\kappa_{B-R}}{\kappa_{B-R}+1}}\;a_{R}\left(u_{B-\mathrm{Ra}\;},v_{B-\mathrm{Ra}\;}\right)\\ \; & {a_{B}}^{T}\left(u_{B-R},v_{B-R}\right)+\sqrt{\rho_{B-R}d_{B-R}^{-\Gamma_{B-R}}\frac{1}{\kappa_{B-R}+1}}{\tilde{H}}_{B-R}, \end{array}$$

where $\rho_{B-R}$, $d_{B-R}$, $\Gamma_{B-R}$ and $\kappa_{B-R}$ represent the reference path loss, the link distance, the path loss exponent, and the Rician factor, respectively. The relevant SAIs are denoted by $u_{B-R}\overset{\Delta }{=}\cos\theta_{B-R}\cos\phi_{B-R}$, $v_{B-R}\overset{\Delta }{=}\cos\theta_{B-R}$, $u_{B-\mathrm{Ra}}\overset{\Delta }{=}\cos\theta_{B-\mathrm{Ra}}\cos\phi_{B-\mathrm{Ra}}$, and $v_{B-\mathrm{Ra}\;}\overset{\Delta }{=}\cos\theta_{B-\mathrm{Ra}}$. Since the established Rician fading channels are dominated by the LoS component, accurately tracking the spatial angles of multi-UAVs is critical for CSI estimation. This will be discussed in Subsection III-A. Here, $\theta_{B-R}$ and $\phi_{B-R}$ represent the elevation and azimuth angles from GBS to IRS, respectively. Correspondingly, $\theta_{B-\mathrm{Ra}}$ and $\phi_{B-\mathrm{Ra}}$ represent the elevation and azimuth angles from IRS to GBS, respectively. ${\tilde{H}}_{B-R}$ represents the random non-line of sight (NLoS) component. The GBS’s antennas and the IRS’s reflective elements are located in the x−z plane. It is assumed that the GBS is linked to the IRS controller via a feedback channel, enabling them to exchange information such as CSI, PSM, and so on. Then, the array responses of GBS and IRS are expressed as

(2) $$\begin{array}{cc} \; & a_{B}\left(u,v\right)={\left[\;1,\ldots ,e^{-j\pi \frac{2d_{GBS}}{\lambda }\left[\left(n_{x}-1\right)u+\left(n_{z}-1\right)v\right]}\right]}^{T}, \end{array}$$

(3) $$\begin{array}{cc} \; & a_{R}\left(u,v\right)={\left[\;1,\ldots ,e^{-j\pi \frac{2d_{IRS}}{\lambda }\left[\left(n_{rx}-1\right)u+\left(n_{rz}-1\right)v\right]}\right]}^{T}, \end{array}$$

where $n_{x}\in \left\{1,\cdots ,N_{x}\right\}$, $n_{z}\in \left\{1,\cdots ,N_{z}\right\}$, $n_{rx}\in \left\{1,\cdots ,N_{rx}\right\}$ and $n_{rz}\in \left\{1,\cdots ,N_{rz}\right\}$. $d_{BS}$, $d_{IRS}$, and λ denote the antenna spacing of GBS, the reflective element spacing of IRS, and the wavelength, respectively. Here, $d_{GBS}$ and $d_{IRS}$ is set as $d_{GBS}=d_{IRS}=\lambda /2$. Correspondingly, the IRS-UA$V_{k}$ and GBS-UA$V_{k}$ links are given by

(4) $$\begin{array}{c} h_{\alpha }^{H}=\sqrt{\rho_{\alpha }d_{\alpha }^{-\Gamma_{\alpha }}\frac{\kappa_{\alpha }}{\kappa_{\alpha }+1}}{a_{R}}^{T}\left(u_{\alpha },v_{\alpha }\right)+\sqrt{\rho_{\alpha }d_{\alpha }^{-\Gamma_{\alpha }}\frac{1}{\kappa_{\alpha }+1}}{\tilde{h}}_{\alpha }^{H}, \end{array}$$

where $\rho_{\alpha }$, $d_{\alpha }$, $\Gamma_{\alpha }$, and $\kappa_{\alpha }$ represent the reference path loss, the link distance, the path loss exponent, and the Rician factor, respectively. ${\tilde{h}}_{\alpha }^{H}$ represents the NLoS component. $\alpha \in \{R-U_{k},B-U_{k}\}$ represents the set of IRS-UA$V_{k}$ and GBS-UA$V_{k}$ links. Notably, the relevant SAI $u_{\alpha }$ and $v_{\alpha }$ are similar to the previous definition. In addition, advanced Doppler shift estimation and compensation techniques are employed to refine the estimation process and ensure robust compensation performance [19,20]. For simplicity, the Doppler shift induced by HSMUAVNs is assumed to be compensated. Moreover, because both the antenna and reflecting elements undergo the same Doppler shift, the SAIs are regarded as immune to the resulting frequency offset [15]. In this paper, we aim to devise an adaptive dual-beam pattern predicated upon the predicted SAI so as to mitigate beam misalignment, augment the performance of the worst-case UAV, and sustain the reliable communication links in HSMUAVNs. This work may be further extended by incorporating more practical IRS-HSMUAVN channel models that account for Doppler shifts, propagation delays, and other factors. The UA$V_{k}$’s received signal can be expressed as

(5) $$\begin{array}{c} y_{k}=h_{B-U_{k}}^{H}\sum_{k=1}^{K}w^{\left(k\right)}s_{k}+h_{R-U_{k}}^{H}\Theta G\sum_{k=1}^{K}w^{\left(k\right)}s_{k}+u_{k}, \end{array}$$

where $u_{k}$ denotes the additive white Gaussian noise, $\sum_{k=1}^{K}w^{\left(k\right)}s_{k}$ represents the signals sent by GBS. Here, $w^{\left(k\right)}\in C^{N\times 1}$ represents the corresponding beamforming vector from GBS to UA$V_{k}$, $s_{k}$ represents the intended data for UA$V_{k}$, and Θ represents IRS’s PSM.

## 3. Adaptive Dual-Beam Tracking Algorithm Design and Optimization

### 3.1. UAVs’ SAI Prediction Model

To fully exploit the temporal dynamics of UAVs, an attention-based double-layer LSTM (DL-LSTM) network [21,22] is employed to predict the multi-UAVs’ SAIs in the PS phase, providing accurate estimates of the beam coverage bound and the required number of beam components. These estimates enable adaptive determination that efficiently avoids beam misalignment. The primary advantage of the attention-based DL-LSTM lies in its ability to construct sequence models that effectively observe the dynamic SAI of multi-UAV. It adeptly captures correlations and dependencies across various time steps, including those from the past, while minimizing the influence of irrelevant and redundant information [22,23]. Note that during the PS, the UAVs can leverage the low-frequency band to feed its current SAI back to the GBS [24]. As shown in Figure 1, the attention-based DL-LTSM-enabled spatial angle prediction model consists of an input layer, recurrent LSTM layer-1, recurrent LSTM layer-2, a fully connected (FC) layer, an output layer, and a attention mechanism. The hyperparameters of the proposed attention-based DL-LTSM-enabled SAI prediction model are shown in Figure 1. The specific description of the attention-based DL-LTSM-enabled SAI prediction model is as follows:

(1) The historical SAI sequence of UAVs are used as the input layer’s data. For example, the past *L*-length SAI sequence of UA$V_{k}$ is denoted as

(6) $$\begin{array}{c} \begin{array}{c} A_{t}^{\left(k\right)}=\left[a_{t-(L-1)*∆t}^{\left(k\right)},\ldots ,a_{t-1*∆t}^{\left(k\right)},a_{t}^{\left(k\right)}\right]=\left[\begin{array}{c} u_{t-(L-1)*∆t}^{\left(k\right)},\ldots ,u_{t-1*∆t}^{\left(k\right)},u_{t}^{\left(k\right)}\\ v_{t-(L-1)*∆t}^{\left(k\right)},\ldots ,v_{t-1*∆t}^{\left(k\right)},v_{t}^{\left(k\right)}, \end{array}\right] \end{array} \end{array}$$

where $a_{t}^{\left(k\right)}={\left[u_{t}^{\left(k\right)},v_{t}^{\left(k\right)}\right]}^{T}$ denotes the SAI of UA$V_{k}$ at time slot *t*. This sequence is fed into a dual-layer LSTM network to extract temporal features. Then, the learned features of multi-UAVs’ SAI in recurrent LSTM layer-2 is denoted as

(7) $$\begin{array}{c} O={\left[o_{1},o_{2},o_{i},\ldots ,o_{S}\right]}^{T}, \end{array}$$

where $o_{i}$ represents the hidden feature vector at the *i*-th time step, and *S* denotes the sliding window size.

(2) The attention mechanism layer receives the learned features sequence O and maps it into query (Q), key (K), and value (V) matrices via learnable weights, which are expressed as

(8) $$\begin{array}{cc} Q & =W_{q}O+B_{q}, \end{array}$$

(9) $$\begin{array}{cc} K & =W_{k}O+B_{k}, \end{array}$$

(10) $$\begin{array}{cc} V & =W_{v}O+B_{v}, \end{array}$$

where $W_{q},W_{k},W_{v}$ and $B_{q},B_{k},B_{v}$ represent the trainable parameters.

(3) Then, the corresponding attention scores, which reflect the significance of the learned features, are computed using the scaled dot-product as

(11) $$\begin{array}{c} \mathrm{Scores}=\frac{QK^{T}}{\sqrt{d}}, \end{array}$$

where *d* denotes the feature dimension. Next, applying softmax yields the attention weights as

(12) $$\begin{array}{c} \alpha =\mathrm{softmax}\left(\frac{QK^{T}}{\sqrt{d}}\right), \end{array}$$

where $\alpha =[\alpha_{1},\alpha_{2},\ldots \alpha_{S}]$ denotes the attention vector. In the following, these weighted features linked to the attention weighting layer are calculated by

(13) $$\begin{array}{c} Z=\alpha ⊗V=\sum_{i=1}^{T}\alpha_{i}v_{i}, \end{array}$$

Here, $V={\left[v_{1},v_{2},\cdots ,v_{S}\right]}^{T}$.

(4) The weighted feature Z is passed to the FC layer and activation function for further processing. Then, the output of the attention-based DL-LSTM SAI prediction model is given by

(14) $$\begin{array}{c} \begin{array}{c} {\tilde{A}}_{t}^{\left(k\right)}=\left[a_{t+1*∆t}^{\left(k\right)},a_{t+2*∆t}^{\left(k\right)},\ldots ,a_{t+L_{s}*∆t}^{\left(k\right)}\right]=\left[\begin{array}{c} u_{t+1*∆t}^{\left(k\right)},u_{t+2*∆t}^{\left(k\right)},\ldots ,u_{t+L_{s}*∆t}^{\left(k\right)}\\ v_{t+1*∆t}^{\left(k\right)},v_{t+2*∆t}^{\left(k\right)},\ldots ,v_{t+L_{s}*∆t}^{\left(k\right)} \end{array}\right]. \end{array} \end{array}$$

Here, $a_{t+L_{s}*∆t}^{\left(k\right)}=g_{\varsigma }\left(Mf_{\lambda }\left(A_{t}^{\left(k\right)}\right)+b\right)$, where $g_{\varsigma }\left(\cdot \right)$, b, and M represent the nonlinear activation function, the weights, and the bias of the FC layer, respectively. Additionally, for the sake of simplicity, $a_{t+L_{s}*∆t}^{\left(k\right)}$ can be expressed as

(15) $$\begin{array}{c} \begin{array}{c} {\tilde{A}}_{t}^{\left(k\right)}=g_{\varsigma }\left(Mf_{\lambda }\left(A_{t}^{\left(k\right)}\right)+b\right)=h_{\phi }\left(A_{t}^{\left(k\right)}\right), \end{array} \end{array}$$

where $h_{\phi }\left(A_{t}^{\left(k\right)}\right)$ represents the general expression of the attention-based DL-LTSM SAI prediction model that contains the parameter ϕ={λ,ς,M,b}. Moreover, during the model training process, we exploit the mean squared error (MSE) cost function to measure the difference between the predicted DL-LSTM spatial angle data and the true data, which is given by

(16) $$\begin{array}{c} \begin{array}{c} G_{MSE}\left(\phi \right)=\left(1/2N\right)\sum_{n=1}^{N}\left|\right|{\hat{A}}_{t}^{(k),n}-h_{\phi }\left(A_{t}^{(k),n}\right)|{|}^{2},n\in \left\{1,2,\cdots ,N\right\}, \end{array} \end{array}$$

where ${\hat{A}}_{t}^{\left(k\right)}=\left[a_{t+1*∆t}^{\left(k\right)},a_{t+2*∆t}^{\left(k\right)},\ldots ,a_{t+L_{s}*∆t}^{\left(k\right)}\right]$. Here, ${\hat{A}}_{t}^{\left(k\right)}$ and $A_{t}^{(k),n}$ denote the label and the input of the *n*-th training example, respectively. Finally, the output layer produced the predicted SAI. The parameter settings of the attention-based DL-LTSM-enabled SAI prediction model are given in Table 1.

### 3.2. Estimation of Beam Coverage Area and Number of Beams per UAV

According to the 3σ criterion [15] and the predicted SAI, the width and length of the adaptive BC area for UA$V_{k}$ can be calculated by

(17) $$\begin{array}{cc} \; & l_{u,t}^{\left(k\right)}=\left|u_{t}^{\left(k\right)}-{\tilde{u}}_{t}^{\left(k\right)}\right|+3\left(\sigma_{tg,u}^{\left(k\right)}+\sigma_{tp,u}^{\left(k\right)}+\sigma_{tm,u}^{\left(k\right)}\right), \end{array}$$

(18) $$\begin{array}{cc} \; & l_{v,t}^{\left(k\right)}=\left|v_{t}^{\left(k\right)}-{\tilde{v}}_{t}^{\left(k\right)}\right|+3\left(\sigma_{tg,v}^{\left(k\right)}+\sigma_{tp,v}^{\left(k\right)}+\sigma_{tm,v}^{\left(k\right)}\right), \end{array}$$

where $\sigma_{tg,u}^{\left(k\right)}\left(\sigma_{tg,v}^{\left(k\right)}\right)$, $\sigma_{tp,u}^{\left(k\right)}\left(\sigma_{tp,v}^{\left(k\right)}\right)$, and $\sigma_{tm,u}^{\left(k\right)}\left(\sigma_{tm,v}^{\left(k\right)}\right)$ represent the standard deviation of the predicted SAI [15], the standard deviation of the process noise, and the measurement standard deviation at UA$V_{k}$, respectively. ${\tilde{u}}_{t}^{\left(k\right)}$ and ${\tilde{v}}_{t}^{\left(k\right)}$ denote the attention-based DL-LTSM predicted SAI in the next time slot, respectively.

In the following, the center of the BC area is denoted by $\left(u_{c,t}^{\left(k\right)},v_{c,t}^{\left(k\right)}\right)$, obtained via $u_{c,t}^{\left(k\right)}=\left(u_{t}^{\left(k\right)}+{\tilde{u}}_{t}^{\left(k\right)}\right)/2,v_{c,t}^{\left(k\right)}=\left(v_{t}^{\left(k\right)}+{\tilde{v}}_{t}^{\left(k\right)}\right)/2.$ To compute the number of beams per UAV tailored to its motion, the length and width of the BC area are divided into $K_{x}$ and $K_{z}$ quantized values, respectively, where $K_{x}=\max\left(N_{x},N_{rx}\right)$, $K_{z}=\max\left(N_{z},N_{rz}\right)$. Thus, the quantized spatial angle on the u-axes and v-axes are expressed as $u_{n}^{\left(k\right)}=-1+\left(2n-1\right)/K_{x},\;n=1,2,3,\ldots ,K_{x}$ and $v_{n}^{\left(k\right)}=-1+\left(2n-1\right)/K_{z},\;n=1,2,3,\ldots ,K_{z}$, respectively. Here, the quantized angle interval are $2/K_{x}$ and $2/K_{z}$. $\Lambda [u_{t}^{\left(k\right)}]$ represents the quantized spatial angle in the $u_{n}^{\left(k\right)}$ that is closest to the $u_{t}^{\left(k\right)}$. Subsequently, the quantified center position of the BC area during a frame are expressed as $u_{\Lambda }^{\left(k\right)}=\Lambda \left[\left(u_{t}^{\left(k\right)}+{\tilde{u}}_{t}^{\left(k\right)}\right)/2\right]$ and $v_{\Lambda }^{\left(k\right)}=\Lambda \left[\left(v_{t}^{\left(k\right)}+{\tilde{v}}_{t}^{\left(k\right)}\right)/2\right]$, respectively, and the quantified BC area from GBS to UA$V_{k}$ is expressed as

(19) $$\begin{array}{c} C_{t}^{\left(k\right)}=\left[\left({u_{t}^{\left(k\right)}}^{-},{u_{t}^{\left(k\right)}}^{+}\right),\left({v_{t}^{\left(k\right)}}^{-},{v_{t}^{\left(k\right)}}^{+}\right)\right], \end{array}$$

where ${u_{t}^{\left(k\right)}}^{-}\left({v_{t}^{\left(k\right)}}^{-}\right)$ and ${u_{t}^{\left(k\right)}}^{+}\left({v_{t}^{\left(k\right)}}^{+}\right)$ denote the lower and upper bounds of the quantized BC on the u(v)-axes, which are given by $u_{t}^{(k)-}=\Lambda \left[u_{\Lambda }^{\left(k\right)}-l_{u,t}^{\left(k\right)}/2\right]$, $u_{t}^{(k)+}=\Lambda \left[u_{\Lambda }^{\left(k\right)}+l_{u,t}^{\left(k\right)}/2\right]$, $v_{t}^{(k)+}=\Lambda \left[v_{\Lambda }^{\left(k\right)}+l_{v,t}^{\left(k\right)}/2\right]$ and $v_{t}^{(k)-}=\Lambda \left[v_{\Lambda }^{\left(k\right)}-l_{v,t}^{\left(k\right)}/2\right]$, respectively. Then, the corresponding number of beam components is $M_{t}^{\left(k\right)}=M_{u,t}^{\left(k\right)}\times M_{v,t}^{\left(k\right)}$, where $M_{u,t}^{\left(k\right)}$ and $M_{v,t}^{\left(k\right)}$ denote the number of quantized points in $\left({u_{t}^{\left(k\right)}}^{-},{u_{t}^{\left(k\right)}}^{+}\right)$ and $\left({v_{t}^{\left(k\right)}}^{-},{v_{t}^{\left(k\right)}}^{+}\right)$, respectively. These values can be calculated by $M_{u,t}^{\left(k\right)}=H\left[u_{t}^{(k)+}\right]-H\left[u_{t}^{(k)-}\right]+1$ and $M_{v,t}^{\left(k\right)}=H\left[v_{t}^{(k)+}\right]-H\left[v_{t}^{(k)-}\right]+1$, respectively. Here, H[·] represents the round operation. Thus, $M_{t}^{\left(k\right)}$ beam components $\left(w_{1}^{\left(k\right)},\cdots ,w_{m_{t}}^{\left(k\right)},\cdots ,w_{M_{t}^{\left(k\right)}}^{\left(k\right)}\right)$ individually point towards each quantized point in the corresponding BC area $C_{t}^{\left(k\right)}$.

By linearly combining the GBS beam components, the transmit beams directed at UA$V_{k}$ and dynamically adapted to its BC boundary are expressed as

(20) $$\begin{array}{c} w_{t}^{\left(k\right)}=\left(1/\sqrt{M_{t}^{\left(k\right)}}\right)\sum_{m_{t}=1}^{M_{t}^{\left(k\right)}}w_{m_{t}}^{\left(k\right)}e^{jm_{t}\beta_{k}}, \end{array}$$

where $\;\beta_{k}\;$ denotes the beamforming parameter that achieves the minimum main lobe variance. Here, the optimal $\;\beta_{k}\;$ can be obtained by utilizing the exhaustive search method. Furthermore, by exploiting 2π-periodic and even function of $\;\beta_{k}\;$, the search space is drastically reduced [25]. Thus, the transmit beamforming matrix of the GBS covering *K* high-speed moving UAVs can be expressed as

(21) $$\begin{array}{c} W_{t}=\left[w_{t}^{\left(1\right)},w_{t}^{\left(2\right)},\ldots ,w_{t}^{\left(k\right)},\ldots ,w_{t}^{\left(K\right)}\right]. \end{array}$$

For the sake of simplicity, we utilize a passive IRS, which offers a clearer and more intuitive perspective. Here, IRS’s PSM is denoted by $\Theta_{t}=\mathrm{diag}\left(\xi_{t}\right)$, where $\xi_{t}={\left[e^{j\vartheta_{1}},\ldots ,e^{j\vartheta_{nr}},\ldots ,e^{j\vartheta_{Nr}}\right]}^{T}$.

### 3.3. Optimization Problem

Maintaining stable channel conditions (e.g., SINR) throughout a frame is essential to prevent disconnection and packet loss. However, sustaining a high SINR becomes extremely challenging in HSMUAVNs, especially for edge UAVs. To provide fair, stable, and reliable communications for HSMUAVNs, the worst-case UAV’s received beam components SINR maximization problem is formulated by jointly designing $w_{m_{t}}^{\left(k\right)}$ and $\xi_{t}$, which is expressed as

(22a) $$\begin{array}{cc} \; & \max_{\left\{w_{m_{t}}^{\left(k\right)},\xi_{t}\right\}}\;\;\min\left(\;\;\;\;\;\gamma_{1}^{\left(k\right)},\ldots ,\;\;\;\;\;\gamma_{m_{t}}^{\left(k\right)},\ldots ,\;\;\;\;\;\;\gamma_{M_{t}^{\left(k\right)}}^{\left(k\right)}\right) \end{array}$$

(22b) $$\begin{array}{cc} \; & \;s.t.\;\sum_{k=1}^{K}\sum_{m_{t}=1}^{M_{t}^{\left(k\right)}}{∥w_{m_{t}}^{\left(k\right)}∥}^{2}\leq P_{GBS}, \end{array}$$

(22c) $$\begin{array}{cc} \; & \begin{array}{cc} \; & 0\leq \vartheta_{n_{r}}\leq 2\pi ,n_{r}\in \{1,\ldots , \end{array}N_{r}\}. \end{array}$$

where $P_{GBS}$ denotes GBS’s transmit power. Then, the received beam components SINR of UA$V_{k}$ is given by

(23) $$\begin{array}{c} \gamma_{m_{t}}^{\left(k\right)}=\frac{{\left|\left(h_{B-m_{t}}^{(k)H}+h_{R-m_{t}}^{(k)H}\Theta_{t}G\right)w_{m_{t}}^{\left(k\right)}\right|}^{2}}{\sum_{k=1}^{K}\sum_{i=1,i\neq m_{t}}^{M_{t}^{\left(k\right)}}{\left|\left(h_{B-m_{t}}^{(k)H}+h_{R-m_{t}}^{(k)H}\Theta_{t}G\right)w_{i}^{\left(k\right)}\right|}^{2}+u_{m_{t}}^{\left(k\right)}}. \end{array}$$

Owing to the strong coupling among variables, problem (22) is non-convex and intractable to solve directly. Therefore, we decouple it into two subproblems (i.e., the GBS’s beam components optimization subproblem and the IRS’s PSM optimization subproblem), and propose a joint optimization algorithm to solve these subproblems iteratively.

### 3.4. GBS’s Beam Components Optimization

Given $\Theta_{t}$, the GBS’s beam components optimization subproblem OP1 is given by

(24a) $$\begin{array}{cc} \; & OP1:\;\max_{\left\{w_{m_{t}}^{\left(k\right)},a\right\}}\;\;a \end{array}$$

(24b) $$\begin{array}{cc} \; & \;s.t.\;\frac{{\left|{\tilde{h}}_{m_{t}}^{\left(k\right)}w_{m_{t}}^{\left(k\right)}\right|}^{2}}{\sum_{k=1}^{K}\sum_{i=1,i\neq m_{t}}^{M_{t}^{\left(k\right)}}{\left|{\tilde{h}}_{m_{t}}^{\left(k\right)}w_{i}^{\left(k\right)}\right|}^{2}+u_{m_{t}}^{\left(k\right)}}\geq a, \end{array}$$

(24c) $$\begin{array}{cc} \; & \;\sum_{k=1}^{K}\sum_{m_{t}=1}^{M_{t}^{\left(k\right)}}{∥w_{m_{t}}^{\left(k\right)}∥}^{2}\leq P_{GBS}, \end{array}$$

where ${\tilde{h}}_{m_{t}}^{\left(k\right)}=\;h_{B-m_{t}}^{(k)H}+h_{R-m_{t}}^{(k)H}\Theta_{t}G$. Let $A_{m_{t}}^{\left(k\right)}={\tilde{h}}_{m_{t}}^{(k)H}{\tilde{h}}_{m_{t}}^{\left(k\right)}$, $B_{m_{t}}^{\left(k\right)}=w_{m_{t}}^{\left(k\right)}w_{m_{t}}^{(k)H}$, and $\Omega_{m_{t}}^{\left(k\right)}=\{B_{1}^{\left(k\right)}$, $\cdots ,B_{m_{t}}^{\left(k\right)},\cdots ,B_{M_{t}^{\left(k\right)}}^{\left(k\right)}\}$. Since ${\left|{\tilde{h}}_{m_{t}}^{\left(k\right)}w_{m_{t}}^{\left(k\right)}\right|}^{2}=\mathrm{Tr}\left(A_{m_{t}}^{\left(k\right)}B_{m_{t}}^{\left(k\right)}\right)$, constraints (24b) and (24c) can be, respectively, rewritten as

(25a) $$\begin{array}{cc} \; & rank(B_{m_{t}}^{\left(k\right)})\leq 1, \end{array}$$

(25b) $$\begin{array}{cc} \; & a\leq \frac{\mathrm{Tr}(A_{m_{t}}^{\left(k\right)}B_{m_{t}}^{\left(k\right)})}{\sum_{k=1}^{K}\sum_{i=1,i\neq m_{t}}^{M_{t}^{\left(k\right)}}\mathrm{Tr}\left(A_{m_{t}}^{\left(k\right)}B_{i}^{\left(k\right)}\right)+u_{m_{t}}^{\left(k\right)}}, \end{array}$$

(25c) $$\begin{array}{c} \sum_{k=1}^{K}\sum_{m_{t}=1}^{M_{t}^{\left(k\right)}}\mathrm{Tr}(B_{m_{t}}^{\left(k\right)})\leq P_{GBS}. \end{array}$$

To satisfy $B_{m_{t}}^{\left(k\right)}=w_{m_{t}}^{\left(k\right)}w_{m_{t}}^{(k)H}$, constraint (25a) needs to be added. However, it makes the subproblem OP1 even trickier. For this reason, constraint (25a) needs to be relaxed by using semi-definite relaxation (SDR) [18]. Additionally, for the constraint (25b), since its right side contains fractional form, it is still a non-convex constraint. Thereby, constraint (25b) needs to be transformed to an equivalent form as follows:

(26) $$\begin{array}{c} a\left(\sum_{k=1}^{K}\sum_{i=1,i\neq m_{t}}^{M_{t}^{\left(k\right)}}\mathrm{Tr}(A_{m_{t}}^{\left(k\right)}B_{i}^{\left(k\right)})+u_{m_{t}}^{\left(k\right)}\right)-\mathrm{Tr}(A_{m_{t}}^{\left(k\right)}B_{m_{t}}^{\left(k\right)})\leq 0. \end{array}$$

Thus, OP1 can be rewritten as

(27a) $$\begin{array}{cc} \; & \max_{\left\{B_{m_{t}}^{\left(k\right)},a\right\}}\;a \end{array}$$

(27b) $$\begin{array}{cc} \; & s.t.\;(25b),(25c). \end{array}$$

Accordingly, problem (27a) is quasi-convex, which can be solved by the bisection method. Finally, $w_{m_{t}}^{\left(k\right)}$ can be found by applying the singular value decomposition (SVD) with the Gaussian randomization [26]. Specifically, by introducing a random vector $z_{1}\in \mathrm{CN}\left(0,1\right)$, the rank-one candidate solution can be written as w˜mt(k)=U∑z1z1z1z1, where U and ∑ can be obtained by using the singular value decomposition (SVD), i.e., $B_{m_{t}}^{\left(k\right)}=U\sum V^{H}$. The randomized solution is tested with (27b) for the feasibility until the objective value of (27a) converges.

### 3.5. IRS’s PSM Optimization

Given $w_{m_{t}}^{\left(k\right)}$, $D_{m_{t}}^{(k)H}=h_{B-m_{t}}^{(k)H}+h_{R-m_{t}}^{(k)H}\Theta_{t}G={\tilde{\xi }}_{t}^{H}C_{m_{t}}^{\left(k\right)}$, where ${\tilde{\xi }}_{t}={\left[\xi_{t}^{T},1\right]}^{H}\;$, $C_{m_{t}}^{\left(k\right)}=\left[diag\left(h_{R-m_{t}}^{(k)H}\right)G;h_{B-m_{t}}^{(k)H}\right]$. Thus, OP2 can be written as

(28a) $$\begin{array}{cc} OP2: & \max_{\left\{\Theta_{t},a\right\}}\;\;a \end{array}$$

(28b) $$\begin{array}{cc} \; & \;s.t.\;\;0\leq \vartheta_{n_{r}}\leq 2\pi ,n_{r}=1,\ldots ,N_{r}, \end{array}$$

(28c) $$\begin{array}{cc} \; & \;\;\;\;\;\;\;\;\frac{{\left|D_{m_{t}}^{(k)H}w_{m_{t}}^{\left(k\right)}\right|}^{2}}{\sum_{k=1}^{K}\sum_{i=1,i\neq m_{t}}^{M_{t}^{\left(k\right)}}{\left|D_{m_{t}}^{(k)H}w_{i}^{\left(k\right)}\right|}^{2}+u_{m_{t}}^{\left(k\right)}}\geq a. \end{array}$$

Let $e_{m_{t}m_{t}}^{\left(k\right)}=C_{m_{t}}^{\left(k\right)}w_{m_{t}}^{\left(k\right)}$, $E_{m_{t}m_{t}}^{\left(k\right)}=e_{m_{t}m_{t}}^{\left(k\right)}e_{m_{t}m_{t}}^{(k)H}$; constraint (28c) can be transformed into

(29) $$\begin{array}{c} \frac{\mathrm{Tr}(E_{m_{t}m_{t}}^{\left(k\right)}{\tilde{\zeta }}_{t})}{\sum_{k=1}^{K}\sum_{i=1,i\neq m_{t}}^{M_{t}^{\left(k\right)}}\mathrm{Tr}\left(E_{m_{t}i}^{\left(k\right)}{\tilde{\zeta }}_{t}\right)+u_{m_{t}}^{\left(k\right)}}\geq a, \end{array}$$

where ${\tilde{\zeta }}_{t}={\tilde{\xi }}_{t}{\tilde{\xi }}_{t}^{H}$. Constraint (29) is then transformed into

(30) $$\begin{array}{c} aE_{\tilde{\zeta }}^{\left(k\right)}-\mathrm{Tr}\left(E_{m_{t}m_{t}}^{\left(k\right)}{\tilde{\zeta }}_{t}\right)\leq 0, \end{array}$$

where

(31) $$\begin{array}{c} E_{\tilde{\zeta }}^{\left(k\right)}=\sum_{k=1}^{K}\sum_{i=1,i\neq m_{t}}^{M_{t}^{\left(k\right)}}\mathrm{Tr}\left(E_{m_{t}i}^{\left(k\right)}{\tilde{\zeta }}_{t}\right)+u_{m_{t}}^{\left(k\right)}, \end{array}$$

and ${\tilde{\zeta }}_{t0}$ represents the feasible value of ${\tilde{\zeta }}_{t}$. Thereby, we can rewrite OP2 as

(32a) $$\begin{array}{cc} \; & \max_{\left\{{\tilde{\zeta }}_{t},a\right\}}\;\;a. \end{array}$$

(32b) $$\begin{array}{cc} \; & \;s.t.\;(12),\;\left(14\right). \end{array}$$

Problem (32) is quasi-convex, and thus, it can be efficiently solved via the bisection method. Additionally, EVD is adopted to obtain $\xi_{t}$ based on ${\tilde{\zeta }}_{t}$.

**Figure 2:**
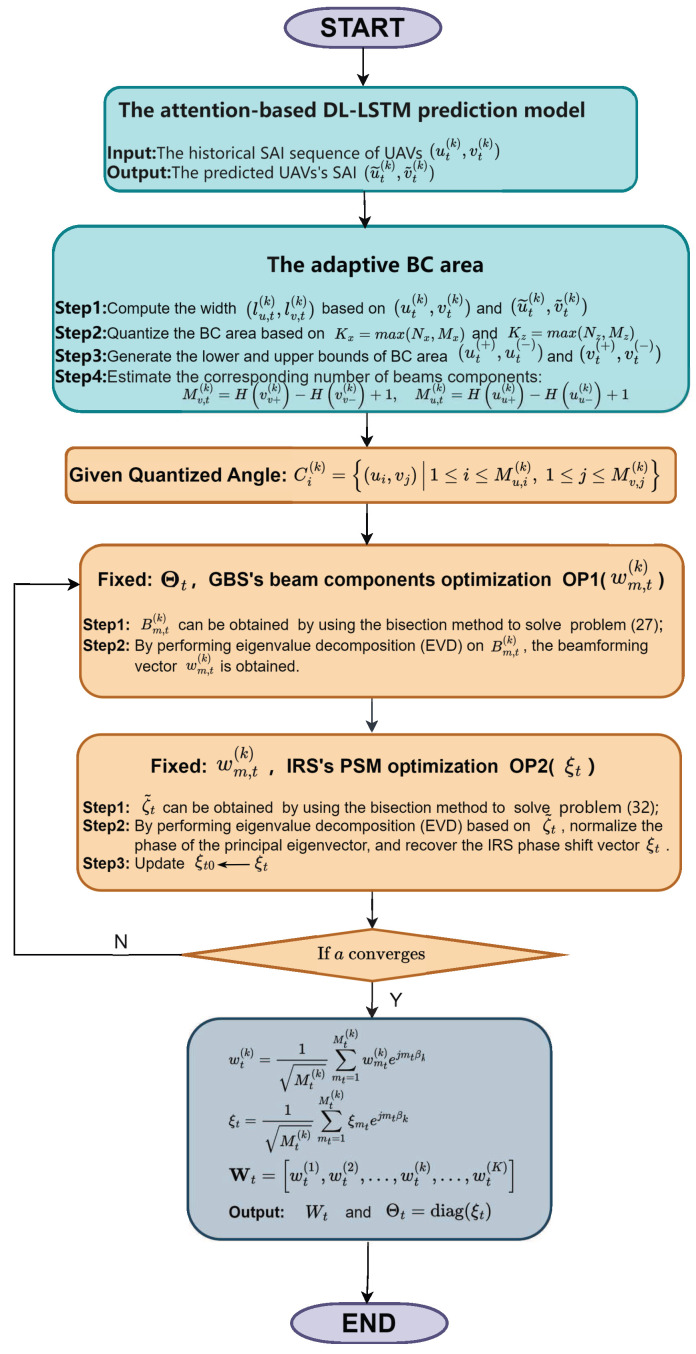
Flowchart of the adaptive dual-beam tracking algorithm.

Algorithm 1 summarizes the overall procedure. Furthermore, Figure 2 depicts a detailed flowchart of the UAV-specific SAI prediction and the iterative optimization process.
**Algorithm 1** Adaptive dual-beam tracking algorithm**Require:** $u_{t}^{\left(k\right)}$, $v_{t}^{\left(k\right)}$, ${\tilde{u}}_{t}^{\left(k\right)}$, ${\tilde{v}}_{t}^{\left(k\right)}$, $\beta_{k}$, G, $h_{R-U_{k}}^{H}$, $h_{B-U_{k}}^{H}$, $P_{GBS}$;1:**Initialize**: $\xi_{t0}$;2:Based on $u_{t}^{\left(k\right)}$, $v_{t}^{\left(k\right)}$, ${\tilde{u}}_{t}^{\left(k\right)}$, ${\tilde{v}}_{t}^{\left(k\right)}$, calculate $l_{u,t}^{\left(k\right)}$, $l_{v,t}^{\left(k\right)}$,$\;M_{t}^{\left(k\right)}\;$;3:**repeat**4:   Given $\xi_{t0}$, update *a* and $B_{m_{t}}^{\left(k\right)}$ by bisection method.5:   **repeat**6:     Given $a=a_{max}/2\in \left[0,a_{max}\right]$, when $B_{m_{t}}^{\left(k\right)}$ exists a feasible domain, the range of *a* has changed to $\left[a_{max}/2,a_{max}\right]$; Otherwise, the range of *a* is $\left[0,a_{max}/2\right]$.7:     According to the range of *a*, the median value of *a* is calculated.8:   **until** the range of *a* is small enough.9:   Obtain $w_{m_{t}}^{\left(k\right)}$ by applying EVD.10:   Utilizing $w_{m_{t}}^{\left(k\right)}$, update *a* and ${\tilde{\zeta }}_{t}$ by bisection method.11:   Obtain $\xi_{t}$ by applying EVD.12:   Update $\xi_{t0}\leftarrow \xi_{t}$.13:**until** convergence14:Calculate $W_{t}=\left[w_{t}^{\left(1\right)},w_{t}^{\left(2\right)},\ldots ,w_{t}^{\left(k\right)},\ldots ,w_{t}^{\left(K\right)}\right]$;**Ensure:** $\;W_{t}\;$,$\Theta_{t}=$ diag$\left(\xi_{t}\right)$.

### 3.6. Complexity Analysis

**Figure 4:**
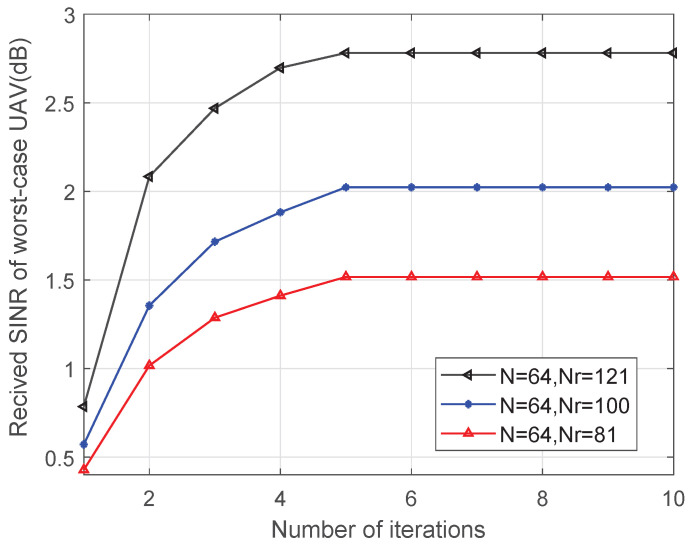
Received SINR of worst-case UAV versus number of iterations.

Next, we analyze the complexity of the proposed joint optimization algorithm for the considered IRS-HSMUAVNs. The overall complexity comprises two primary parts. Specifically, since subproblem OP1 is solved by the bisection method and the EVD, its complexity is $O\left(log\left(a/\varepsilon_{op1}\right)+{\left(N+1\right)}^{3}\right)$. Similarly, the subproblem OP2 is a non-convex problem which adopts the same solving method as OP1, so the complexity used for computing IRS’s PSM is $O\left(log\left(a/\varepsilon_{op2}\right)+{\left(N_{r}+2\right)}^{3}\right)$. Thus, the overall complexity of the proposed joint optimization algorithm is

(33) $$\begin{array}{c} O\left(Iter(log\left(a/\varepsilon_{op1}\right)+log\left(a/\varepsilon_{op2}\right)+{\left(N+1\right)}^{3}+{\left(N_{r}+2\right)}^{3})\right), \end{array}$$

where Iter denotes the required iteration numbers, $\varepsilon_{op1}$ and $\varepsilon_{op2}$ denote tolerance level. Note that the complexity of linearly weighting the obtained beam components is very low, almost negligible. Figure 4 demonstrates that the proposed algorithm achieves rapid convergence.

## 4. Simulation Results

In this section, simulation results are provided to illustrate the performance of the proposed adaptive dual-beam tracking scheme for IRS-assisted HSMUAVNs. According to [15,27,28], the related simulation parameters are given as follows. For simulations, we consider that the speeds of UAVs are randomly determined in U [170, 200] [km/hr], where U represents uniform distribution. The number of UAVs *K* is set to 3. $\sigma_{tp,u}=\sigma_{tp,v}=5\cdot 10^{-3}$ and $\sigma_{tm,u}=\sigma_{tm,v}=10^{-3}$. The initial location of each UAV is randomly generated in a space with a radius of 120 m around GBS. The location of GBS is given by (0 m, 0 m, 0 m). And the location of IRS is given by (0 m, −50 m, 50 m). Additionally, the Rician factor $\kappa_{B-I}=\kappa_{I-U_{k}}=10$ dB, $\kappa_{B-U_{k}}=5$ dB, and the path loss exponent $\chi_{B-I}=\chi_{I-U_{k}}=2$ dB, $\chi_{B-U_{k}}=2.2$ dB. The time slot length ∆t=20 ms and $L_{s}=3$. The neural network is retrained at every 60 ms.

**Figure 3:**
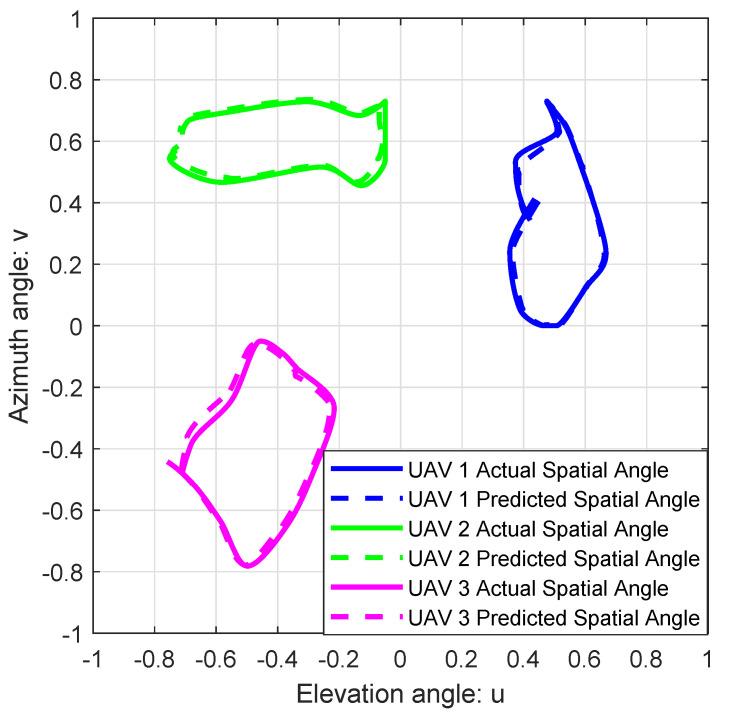
High-speed multi-UAVs trajectories in u-v plan.

Figure 3 shows the comparison between the actual spatial angles and the predicted spatial angles of high-speed multi-UAVs. Although the UAVs move at high speeds, it can be observed from Figure 3 that the spatial angle prediction error for each UAV remains consistently minimal at every moment. From Figure 3, the average displacement errors (ADE) between the actual and the predicted SAIs of UA$V_{1}$, UA$V_{2}$, and UA$V_{3}$ are 0.0147, 0.009, and 0.0143, respectively. Here, ADE denotes the average Euclidean distance between the actual and the predicted SAIs. Therefore, by utilizing the attention-based DL-LSTM, the GBS can effectively predict the spatial angles of high-speed multi-UAVs.

Figure 4 depicts the proposed algorithm’s convergence performance when N=64, $N_{r}=\left\{81,100,121\right\}$ when $P_{GBS}=30$ dB. It is shown that the worst-case UAV’s received SINR can converge fast within five iterations. Furthermore, the worst-case UAV’s received SINR performance enhances as $N_{R}$ increases.

**Figure 5:**
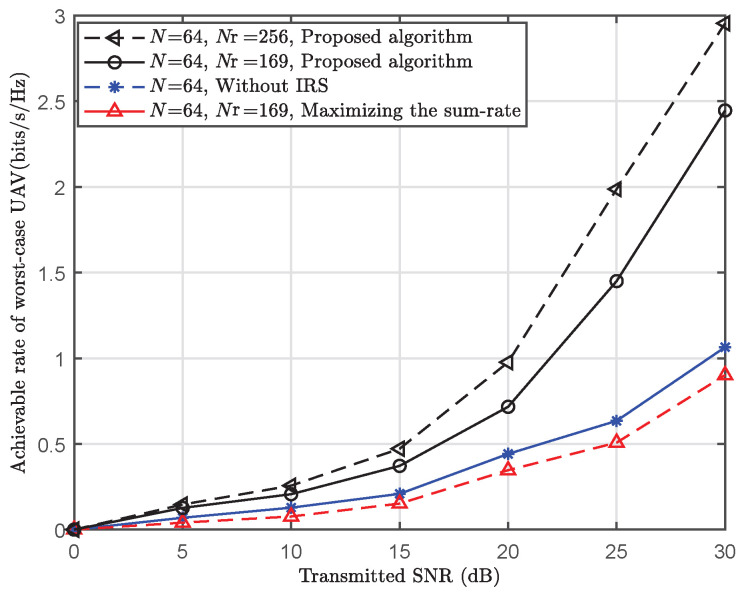
Achievable rate of worst-case UAV versus transmitted SNR (t=35 ms).

Figure 5 shows the worst-case UAV’s achievable rate versus the transmitted SNR under different schemes. Here, “**Maximizing the sum-rate with IRS** ” refers to UAVs’ sum-rate maximization by jointly optimizing GBS’s beamforming and IRS’s PSM. “**Without IRS**” refers to the proposed beam tracking algorithm for GBS-UAV communications without the assistance of the IRS. As shown in Figure 5, compared with “**Maximizing the sum-rate with IRS**”, the proposed algorithm improves the achievable rate of the worst-case UAV by 170% when the transmitted SNR =30 dB, *N* = 64, and $N_{r}$ = 169. This is because “**Maximizing the sum-rate with IRS**” benefits UAVs in good channel conditions but harms the worst-case UAV. Thus, such unfair resource allocation leads to a low achievable rate for the worst-case UAV. Additionally, the worst-case UAV’s performance of “**Without IRS**” is obviously worse than that of the proposed algorithm. The major reason is that IRS offers more DoFs. Therefore, IRS’s resources are beneficial for enhancing the communication capability of the worst-case UAV under the max–min fairness beam design scheme.

**Figure 6:**
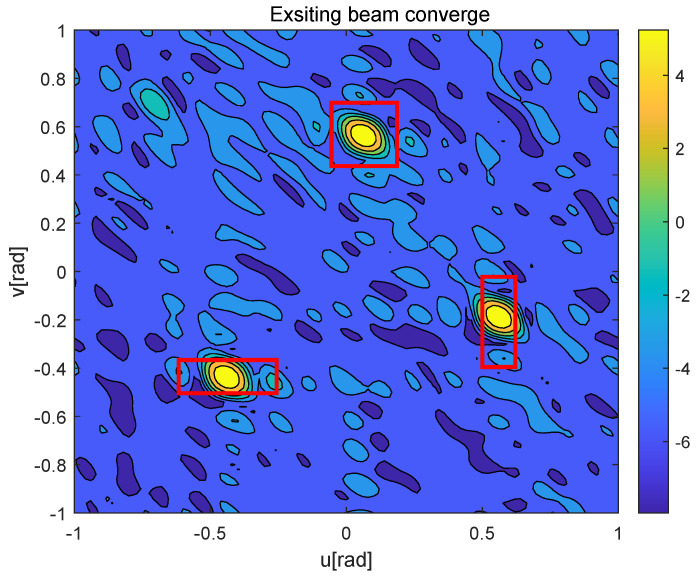
Exsiting beam converge of multi-UAVs.

**Figure 7:**
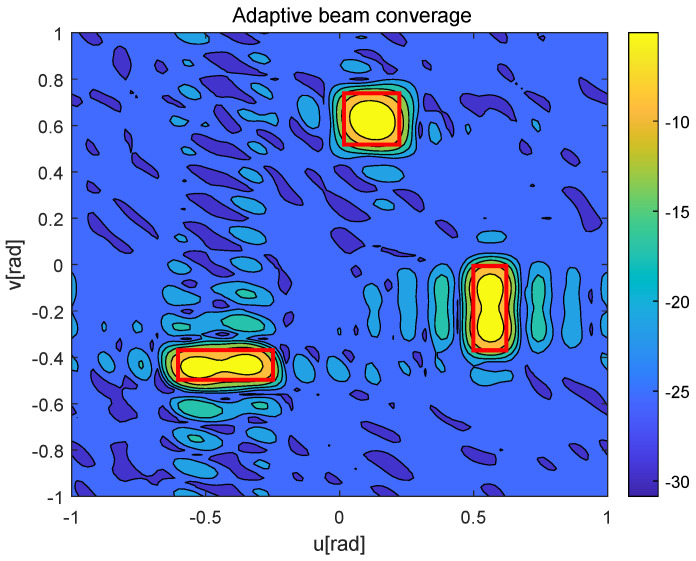
Adaptive beam converage of multi-UAVs.

Figure 6 and Figure 7 depict the beamforming gain [dBm] of the existing beam tracking algorithm [15] and the proposed beam tracking algorithm, respectively. Here, the red boxes represent the BC areas corresponding to each moving UAV during a frame. As shown in Figure 6, the existing beam tracking algorithm suffers from limited coverage area when aligning with high-speed UAVs, failing to accommodate their full mobility range. This inherent limitation consequently leads to unstable communication performance and frequent link interruptions. In contrast, the proposed algorithm generates adaptive dual-beam coverage that maintains a precise tracking of each UAV’s movement, as demonstrated in Figure 7, thereby ensuring stable and high-quality service for all mobile UAVs.

**Figure 8:**
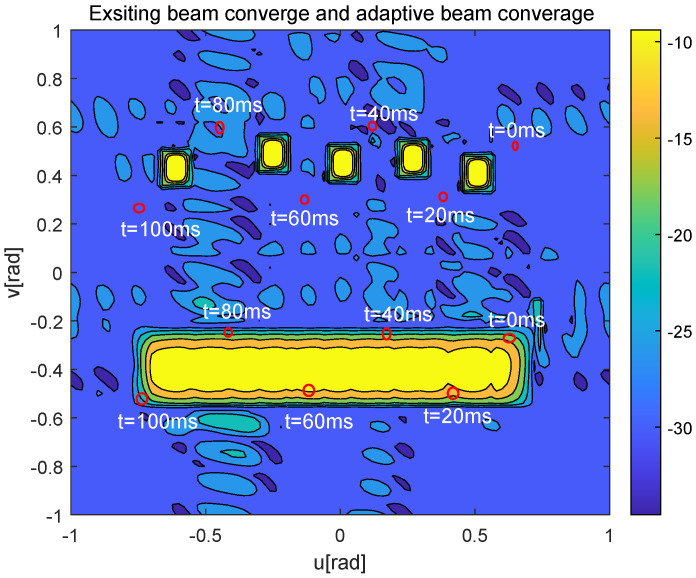
Contour map of beamforming gain of the exsiting beam tracking algorithm and the proposed adaptive beam tracking algorithm, when *N* = 64, $N_{r}$ = 64, SNR = 10 dB.

Figure 8 further shows the worst-case UAV’s beam gain, where the red boxes represent the BC area corresponding to each moving UAV, and the red dots represent the SAI of the worst-case UAV at the corresponding time t={0,20,40,60,80,100} ms. From Figure 8, the existing algorithm only provides a good beam gain in the central point of the current and the next prediction moment of the worst-case UAV, but the beam gain decreases sharply in nearby areas. In contrast, the beam generated by the proposed algorithm always points to the potential moving area of the worst-case UAV.

**Figure 9:**
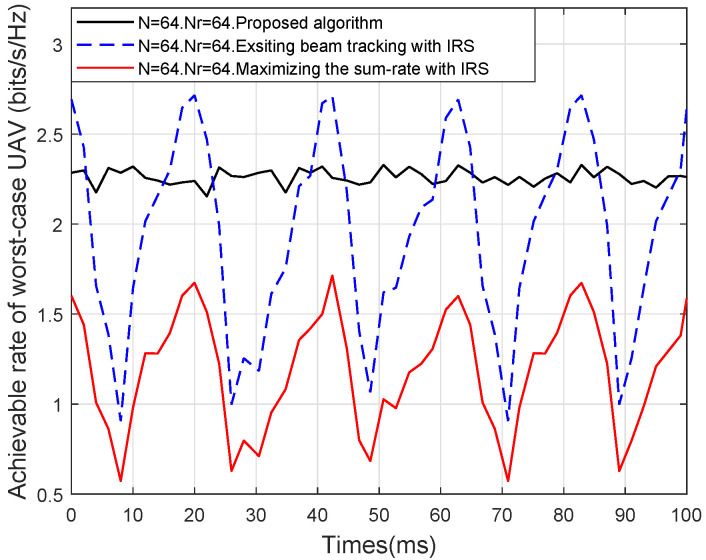
Achievable rate of worst-case UAV versus time.

To more intuitively show the beam tracking performance, the worst-case UAV’s achievable rate versus time when $P_{GBS}=30$ dB is presented in Figure 9. As shown in Figure 9, despite the help of IRS, “**Existing beam tracking**” outperforms the proposed algorithm at narrow beam alignment time, but its performance drops dramatically at other times, thereby easily resulting in link disconnection. At the same time, the performance of “**Maximizing the sum-rate with IRS**” also performs poorly and is instable compared with the other algorithms, since it benefits UAVs in favorable channels yet disadvantages those experiencing the worst-case CSI conditions. In contrast, the proposed algorithm always sustains the stable and robust performance of the worst-case UAV during a frame, since the worst-case UAV is given priority in the resources allocation under the min–max optimization framework. Consequently, the proposed adaptive dual-beam tracking design achieves a well-balanced resource allocation, which is vital for ensuring fair, stable, and reliable communications in IRS-HSMUAVNs.

## 5. Conclusions

In this paper, we studied a novel adaptive dual-beam tracking scheme in an adaptive mode for IRS-assisted HSMUAVNs. An attention-based DL-LSTM neural network was employed to estimate the BC and the beam components of the optimal beam direction for each high-speed UAV. Furthermore, we formulated a worst-case UAV’s received beam components SINR maximization problem by jointly optimizing the beam components at the GBS and the PSM of IRS. To solve this optimization problem, we decouple it into two subproblems and iteratively addressed them using the semi-definite relaxation, bisection method, and eigenvalue decomposition techniques. Simulation results demonstrated that the proposed algorithm not only enhanced the worst-case UAV’s performance but also maintained stable and robust communications throughout each frame. Furthermore, these results highlighted the critical importance of adaptive dual-beam tracking design in ensuring reliable communications across the entire HSMUAVNs. In future work, we will address the extension of channel tracking to a more realistic IRS-assisted HSMUAVN channel.

## Figures and Tables

**Table 1:** The attention-based DL-LTSM-enabled SAI prediction model.

Hyperparameter Name	Numerical Value
Input size of $A_{t}^{k}$	2×L
Size of $z_{i}^{l_{1}}$ in LSTM layer-1	64×1
Size of $z_{i}^{l_{2}}$ in LSTM layer-2	64×1
Attention layer size of Query/Key/Value	64×64
FC layer activation function	Linear
Dropout Rate	0.2
Learning Rate	0.001
Epochs	500
Batch size	128
Output size of $a_{t+{∆}_{t}}^{k}$	$2\times T_{s}$

## Data Availability

No new data were created or analyzed in this study.
